# Thermally promoted addition of undecylenic acid on thermally hydrocarbonized porous silicon optical reflectors

**DOI:** 10.1186/1556-276X-7-311

**Published:** 2012-06-19

**Authors:** Tero Jalkanen, Ermei Mäkilä, Tetsuo Sakka, Jarno Salonen, Yukio H Ogata

**Affiliations:** 1 Institute of Advanced Energy, Kyoto University, Uji, Kyoto, 611-0011, Japan; 2 Department of Physics and Astronomy, University of Turku, Turku, FI-20014, Finland; 3 Turku University Centre for Materials and Surfaces, University of Turku, Turku, FI-20014, Finland

**Keywords:** Porous silicon, Interference filter, Optical microcavity, Chemical stability

## Abstract

Thermally promoted addition of undecylenic acid is studied as a method for modifying porous silicon optical reflectors that have been pre-treated with thermal hydrocarbonization. Successful derivatization of undecylenic acid is demonstrated and confirmed with Fourier transform infrared and X-ray photoelectron spectroscopies. The results indicate that the hydrocarbonization pre-treatment considerably improves stability against oxidation and chemical dissolution in basic environments. The two-step treatment also does not cause an appreciable change on sample reflectance spectra, which enables the use of the functionalized structures in optical sensing applications.

## Background

The use of nanostructured porous silicon (PSi) in biosensing applications has received a lot of attention in recent years [1-5]. This interest is, for the most part, due to the large internal surface area and easily functionalizable surface of PSi [6]. In addition, fabrication of PSi with electrochemical anodization is a straightforward process that enables control over the pore size and morphology of the resulting material. The favorable optical properties of PSi also enable the preparation of interference filters and optical thin films [7]. Furthermore, PSi-based thin films and optical filters have been used in gas and biosensing applications [8,9]. We also need to mention 1/f noise in polymorphous silicon films [10,11].

The surface of freshly prepared PSi is covered with hydride species and can therefore be functionalized by grafting molecules, with functional groups, on the surface [12,13]. This can be achieved, for example, with thermal hydrosilylation of undecylenic acid [14]. Even though the stability of modified PSi in basic solutions, such as aqueous KOH, is considerably increased, the surface coverage is not complete, and hydrides remain on the surface [15,16]. These un-reacted Si-H bonds on PSi surface are susceptible to oxidation, and furthermore, oxidized surface slowly dissolves in aqueous basic solutions [2,17]. This phenomenon has been linked with baseline drift of PSi-based biosensors, leading to non-reproducible results [17]. More complete surface passivation may be obtained with the thermal hydrocarbonization (THC) treatment [18]. Moreover, the utilization of THC-treated PSi optical filters, in gas sensing applications, has already been demonstrated [19]. Lately, it has also been shown that the THCPSi surface may also be further functionalized by attaching molecules with a functional group to the hydrocarbonized surface [20,21].

In this work, we evaluate the effects of thermally promoted undecylenic acid addition on THCPSi optical reflectors. Successful functionalization of the hydrocarbonized surface is verified with Fourier transform infrared (FTIR) and X-ray photoelectron spectroscopies (XPS). The stability of the modified THCPSi surface is tested under several high-pH environments and compared to as-anodized PSi and PSi treated with thermal hydrosilylation of undecylenic acid. A large improvement in the stability is observed, which indicates that the new two-step treatment is promising for producing optical biosensors.

## Methods

### Sample preparation

Porous silicon-based optical microcavities and rugate filters were prepared by anodizing single-crystal boron-doped *p*-type Si wafers ((100) 0.005 to 0.03 *Ω*cm) in an electrolyte solution composed of HF (46-48 wt.%) and ethanol in 1:1.7 ratio (*v*/*v*). The anodization current was controlled with a programmable current source (Keithley 6221, Keithley Instruments Inc., Cleveland, OH, USA). Sinusoidal anodization current with current density values oscillating between 63.3 and 113.9 mA/cm^2^ was used. The sine wave period length was 5 s, and the total anodization time was 210 s. A cavity layer was added in the middle by applying 63.3 mA/cm^2^ of constant current density for 12.5 s. Samples were prepared in a single-tank electrochemical cell, which left an area of 0.79 cm^2^ of the Si electrode exposed to the electrolyte. InGa eutectic was applied to the backside of the Si electrodes to ensure an ohmic contact with the Cu plate, which was used as a current collector. Prior to the anodization, the Si samples were cleaned with successive ultrasonic treatments in acetone and ultrapure water (Millipore, Milli-Q Gradient, Millipore Co., Billerica, MA, USA) and finally dipped in HF (5 wt.%) to remove surface oxides.

### Chemical modifications

Thermal hydrocarbonization was performed on dried samples in a quartz tube [4]. After a 15-min acetylene treatment at 500°C, the samples were cooled down to room temperature under nitrogen flow. The hydrocarbonized porous Si samples were immersed in neat undecylenic acid solution for 16 h at 120°C, which resulted in partial carboxyl group termination [21].

Derivatization of undecylenic acid was also performed on as-anodized PSi. After anodization, the dried samples were inserted into a round bottom flask and immersed in neat undecylenic acid solution. The flask was then connected to a Schlenk line and purged with three vacuum-refill cycles. The samples were treated under argon atmosphere for 20 h at 120°C. After both treatments, the functionalized samples were copiously rinsed with dichloromethane, tetrahydrofuran, and ethanol to remove excess undecylenic acid.

### Characterization

Reflectance measurements were conducted with an Ocean Optics HR4000CG-UV-NIR spectrometer (Ocean Optics, Inc., FL, USA), which was connected to a tungsten halogen lamp (LS-1, Ocean Optics). FTIR spectra were recorded with a Perkin Elmer Spectrum BX spectrometer (PerkinElmer, Waltham, MA, USA) using an attenuated total reflectance (ATR) accessory (MIRacle, Pike Technologies Inc., Madison, WI, USA). Microcavity structure was examined with scanning electron microscopy (JEOL JSM-6500FE, JEOL Ltd., Akishima, Tokyo, Japan). XPS (JEOL JPS-9010MC) was used for analyzing the surface of the chemically modified microcavities. Al K_*α*_ line (1,486.6 eV) was used as an X-ray source and operated at 10 kV and 10 mA (100 W). The samples were kept in high vacuum up to 10^-7^ Pa during the XPS measurements. Binding energies were referenced to adventitious carbon C 1s signal at 284.5 eV. Thermogravimetric measurements (TGA 7, Perkin Elmer) were used for assessing the thermal stability of the samples.

### Stability experiments

Methylamine (MA) (mixed in water, 40%) vapor measurements were conducted in an atmospheric chamber equipped with an optical window. Vapor concentration inside the chamber was controlled by adjusting relative gas-flow ratios with mass-flow controllers. Nitrogen was used as carrier gas [19]. Gas-liquid chromatography (GLC) (GC-14B, Shimadzu Corporation, Nakagyo-ku, Kyoto, Japan) was used for determining the MA concentration. Concentration was determined by directing the gas stream (nitrogen and MA) inside 20 mL of 1.0 M HCl aqueous solution for 1 min. Phenolphthalein solution (0.1%; approximately 90% ethanol, 200 *μ*L) and isopropanol (internal reference, 42.0 *μ*L, 0.500 mmol) were added to the solution. Back titration was performed with NaOH (1.0 M) aqueous solution and was used for confirming the results. NaOH solution was added slightly in excess (*ca* 21 mL), and the solution was analyzed with GLC.

## Results and discussion

### Changes in the chemical composition

**Figure 1:**
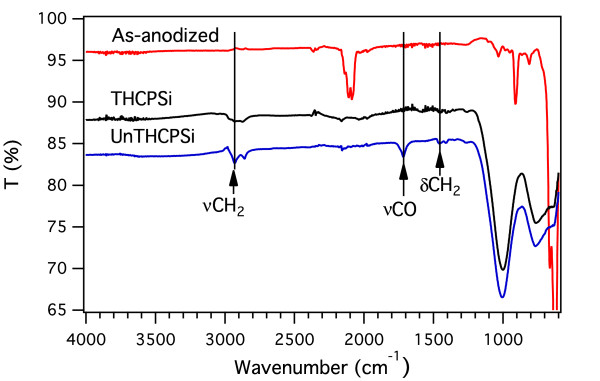
**FTIR spectra.** ATR-FTIR spectra for as-anodized PSi, THCPSi, and UnTHCPSi microcavities.

**Figure 2:**
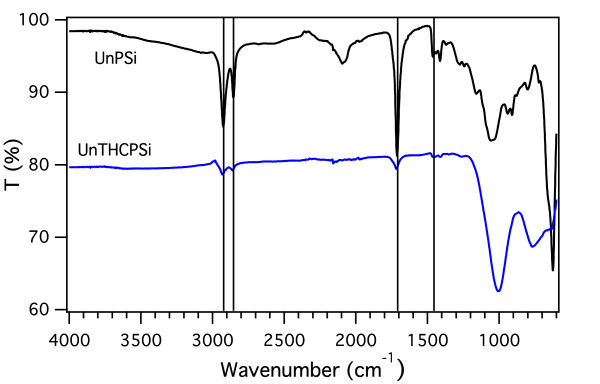
**Comparison of FTIR spectra.** ATR-FTIR spectra for UnPSi and UnTHCPSi microcavities. The features related to the binding of undecylenic acid on the surface appear in the same regions.

The gradual change in the surface chemistry of the PSi structure during the UnTHC treatment is clearly seen in the FTIR spectra (Figure 1). Spectrum recorded for the as-anodized sample displays peaks related to stretching of hydride species at 2,100 cm^-1^. Peaks caused by SiH_2_ scissor bending and wagging are observed at 916 and 628 cm^-1^, respectively [22]. SiH bending at 667 cm^-1^ is also visible [22]. After thermal hydrocarbonization, these peaks disappear and are replaced with strong peaks around 1,000 cm^-1^ arising from the hydrocarbon termination [18]. In addition, smaller peaks related to hydrocarbon species are observed at 2,950 cm^-1^. As the hydrocarbon surface is functionalized with undecylenic acid, emergence of three new peaks is clearly observed (Figure 1). Peaks due to the stretching and deformation of CH_2_ of the alkyl chain appear at 2,922 and 1,460 cm^-1^, respectively [14]. The absorption peak at 1,712 cm^-1^ is attributed to the carbonyl stretching (*ν*CO) and indicates a partial carboxyl termination of the UnTHC-treated surface. Comparison between the FTIR spectra recorded for the UnTHCPSi and UnPSi-treated microcavities shows that the peaks related to successful addition of undecylenic acid on the surface appear exactly at same wavenumber regions for both samples (Figure 2). Although the exact chemical reaction mechanism is not clear, thermogravimetry measurements clearly show the strong thermal stability of UnTHCPSi and rule out the possibility of mere physisorption [see Figure S1 in Additional file 1.

**Figure 3:**
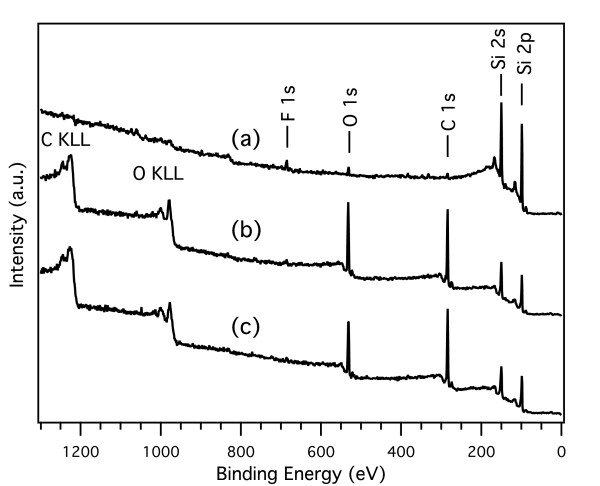
**XPS survey spectra.** XPS survey spectra measured for as-anodized PSi **(a)**, freshly prepared UnPSi **(b)** and UnTHCPSi **(c)** microcavities.

The differences in chemical composition are also clearly seen in the XPS survey spectra recorded for as-anodized PSi, UnPSi, and UnTHCPSi microcavities (Figure 3). As-anodized PSi shows clear peaks for Si, whereas only trace amounts of C, F, and O are observed (Figure 3a). An increase in carbon and oxygen contents can be observed in the spectra recorded for UnPSi and UnTHCPSi (Figure 3b,c, respectively). This is seen as strengthening of the C 1s and O 1s peaks as well as the appearance of Auger peaks. The C 1s core level spectra also reveal a small peak at an approximate binding energy of 289.5 eV, which is characteristic to the carboxyl group [14,23].

### Effects of chemical modification on PSi optical reflectors

**Figure 4:**
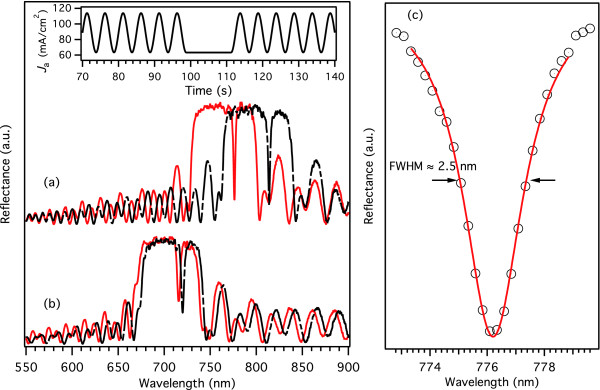
**Effect of surface modification on reflectance spectra.** The effect of thermal hydrosilylation of undecylenic acid (**a**) and the UnTHC treatment (**b**) on the reflectance spectra of PSi-based graded-index microcavities. The solid red line represents spectra for as-anodized samples, and the dashed black line is the spectrum recorded after modification. The inset shows the anodization current profile as a function of anodization time. The constant current region results in a cavity layer that is created between two rugate filters. Full width at half maximum (FWHM) values of the cavity resonance, determined from a Lorentzian fit to experimental data, show values in the order of a few nanometers (**c**).

Effects of the chemical modifications on the reflectance spectra of PSi microcavities are shown in Figure 4. Both treatments, namely thermal hydrosilylation of undecylenic acid (UnPSi) and thermally promoted addition of undecylenic acid on thermally hydrocarbonized porous silicon (UnTHCPSi), resulted in a shift of the reflectance spectrum. The UnPSi spectra showed a redshift that was usually in the order of 20 to 38 nm (Figure 4a), whereas the UnTHC treatment usually resulted in a redshift in the order of 1 to 10 nm (Figure 4b). The exact value of the spectral shift varied between samples for both chemical modifications, indicating that the amount of undecylenic acid bound on the surface is quite sensitive to slight changes in experimental conditions. The redshift recorded for the UnPSi is related to the covalent attachment of the undecylenic acid molecule on the inner surface of the porous structure. The UnTHC treatment is a two-step process where the thermal hydrocarbonization causes a blueshift in the order of 10 nm followed by a successive redshift as the undecylenic acid binds to the THCPSi surface [23].

**Figure 5:**
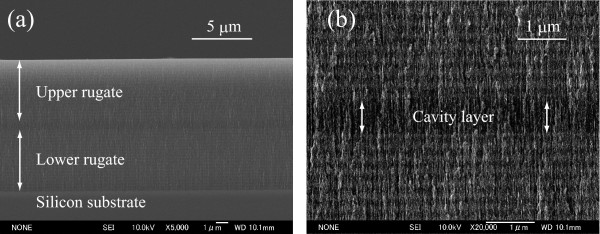
**Scanning electron micrograph.** Cross-sectional scanning electron micrographs showing the UnTHCPSi microcavity structure (**a**) and a magnification of the cavity layer (**b**).

The shape of the reflectance spectra remains virtually unchanged for both UnPSi and UnTHCPSi microcavities (Figure 4). Moreover, the quality of the cavity resonance, which is very delicate to slight changes in the structure, was not affected. The best FWHM values recorded for the cavities were smaller than 2.5 nm (Figure 4c). Figure 5 shows a cross-sectional scanning electron micrograph of an UnTHC-modified PSi microcavity. It is obvious that the cavity structure is preserved after the treatment, and well-defined interfaces can be observed between the cavity layer and the upper and lower filter layers (Figure 5b).

### Stability under basic environments

**Figure 6:**
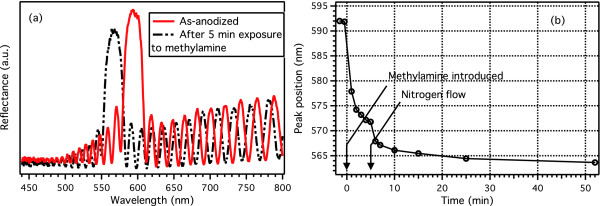
**As-anodized rugate filter response to methylamine.** Reflectance spectra for an as-anodized PSi rugate filter measured before and after 5-min exposure to 10.7% (volume) of methylamine vapor (**a**). The change in the reflective peak position plotted as a function of exposure and recovery time (**b**). The instantaneous blueshift indicates rapid oxidation of the PSi optical filter.

As-anodized PSi is highly unstable under basic conditions. This is demonstrated in Figure 6, where a clear blueshift in the reflectance spectrum of a PSi rugate filter is observed after just 5-min exposure to 10.7% of MA vapor. Usually when vapor adsorbs inside a PSi optical reflector, a redshift, connected to an increase in the effective refractive index, is observed [8]. However, in this case, the MA vapor creates highly basic conditions inside the porous matrix resulting in rapid oxidation. This is seen as an instantaneous blueshift, which increases in accordance with exposure time (Figure 6b). After 47-min recovery period, an irreversible blueshift close to 30 nm is observed. When the same experiment is performed for a UnPSi rugate filter, the filter displays a redshift upon MA adsorption. A blueshift in the order of 2 nm is observed after the 47-min recovery period, indicating slight oxidation. The UnTHCPSi rugate filter also displays a redshift when exposed to MA vapor, but unlike the as-anodized PSi and UnPSi filters, no blueshift is observed. This suggests that sample stability against oxidation is increased, as discussed in reference [23].

**Figure 7:**
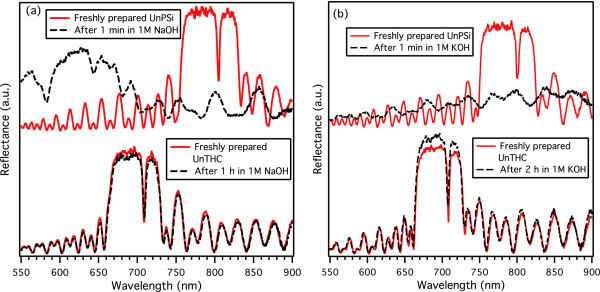
**Aqueous stability.** Reflectance spectra measured for freshly prepared UnPSi and UnTHC microcavities, and spectra measured after immersion in 1 M aqueous NaOH (**a**) and KOH (**b**). The UnPSi microcavities show clear dissolution after 1-min immersion, whereas the spectra measured for UnTHCPSi microcavities show only minor changes after 1- and 2-h immersion.

In order to compare the stability of differently modified surfaces in aqueous solutions, as-anodized PSi, UnPSi, and UnTHCPSi microcavities were immersed in 1 M solutions of aqueous NaOH and KOH. The as-anodized samples dissolved within 30 s in both NaOH and KOH solutions. The UnPSi microcavities displayed considerably improved stability, and complete dissolution was observed after 2.5 min. Unlike the as-anodized microcavities and microcavities treated with thermal hydrosilylation of undecylenic acid, the UnTHC-treated microcavities did not display prominent bubble formation upon immersion to NaOH or KOH. Figure 7 shows the reflectance spectra measured for freshly prepared UnPSi and UnTHCPSi microcavities and the spectra after immersion to aqueous NaOH (Figure 7a) and KOH (Figure 7b). The spectra for UnPSi cavities show clear dissolution after only 1-min immersion. In stark contrast, the spectra measured for UnTHC cavities show only minor changes even after 1-h immersion to NaOH and 2-h immersion to KOH. Moreover, the position of the cavity resonance has remained virtually unchanged. Based on the stability exhibited by UnTHCPSi optical reflectors and also taking into account the possibility for further surface functionalization of the UnTHCPSi structure [see Figure S2 in Additional file 1], we can conclude that the UnTHCPSi reflectors show promise for utilization in biosensing applications.

After 24 h of immersion, the UnTHC-treated microcavity had completely dissolved in KOH. The sample immersed in NaOH had still not completely dissolved, but the porous layer had become detached from the substrate, and broke into fragments as we tried to remove it from the beaker. The slight differences observed for dissolution in NaOH and KOH are attributed to experimental error. Immersion times required for complete dissolution of differently modified microcavities are gathered in Table 1.

**Table 1:** Immersion time needed for dissolving PSi microcavities in aqueous solutions


**Solution**	**1 M NaOH**	**1 M KOH**
As-anodized PSi	30 s	30 s
UnPSi	<3 min	<3 min
UnTHC	24 h	24 h

## Conclusions

Undecylenic acid addition to thermally hydrocarbonized PSi was demonstrated. The incorporation of the molecule on the THCPSi surface was verified with FTIR and XPS measurements. In addition, UnTHC treatment has only minor effects on the optical quality of PSi-based interference reflectors, and even delicate structures such as optical microcavities can be modified with the UnTHC treatment. UnTHCPSi showed remarkable improvement in stability when compared to as-anodized PSi and UnPSi. The improved stability under aqueous basic solutions suggests that the functionalized surface would be useful in developing optical biosensors.

## Competing interests

The authors declare that they have no competing interests.

## Authors’ contributions

TJ and EM designed the experiments and analyzed the data. TJ prepared the PSi samples, performed most of the experiments, and wrote the first draft of the manuscript. EM made the UnTHC surface modifications and performed the FTIR measurements. TS, JS, and YHO gave advice on technical details. All authors read and approved the final manuscript.

## Supplementary Material

Additional file 1 **Figures S1 and S2.** Thermogravimetric measurements and FTIR spectra. Click here for file
