# Supplementary material for: Thermally promoted addition of undecylenic acid on thermally hydrocarbonized porous silicon optical reflectors
Source: Nanoscale Res Lett. 2012 Jun 19;7(1):311. doi: 10.1186/1556-276X-7-311 (PMC3431257; doi:10.1186/1556-276X-7-311)
Supplement: Additional file 1 — Figures S1 and S2. Thermogravimetric measurements and FTIR spectra. [file 1556-276X-7-311-S1.pdf]

Supplementary data:

Thermally promoted addition of undecylenic acid on thermally hydrocarbonized porous silicon optical reflectors

By Tero Jalkanen[1,2] , Ermei Mäkilä[2] , Tetsuo Sakka[1] , Jarno Salonen[2,3], and Yukio H. Ogata[1]

[1]Institute of Advanced Energy, Kyoto University, Uji, Kyoto 611-0011, Japan

[2]Department of Physics and Astronomy, University of Turku, FI-20014 Turku, Finland

[3]Turku University Centre for Materials and Surfaces, University of Turku, FI-20014 Turku, Finland

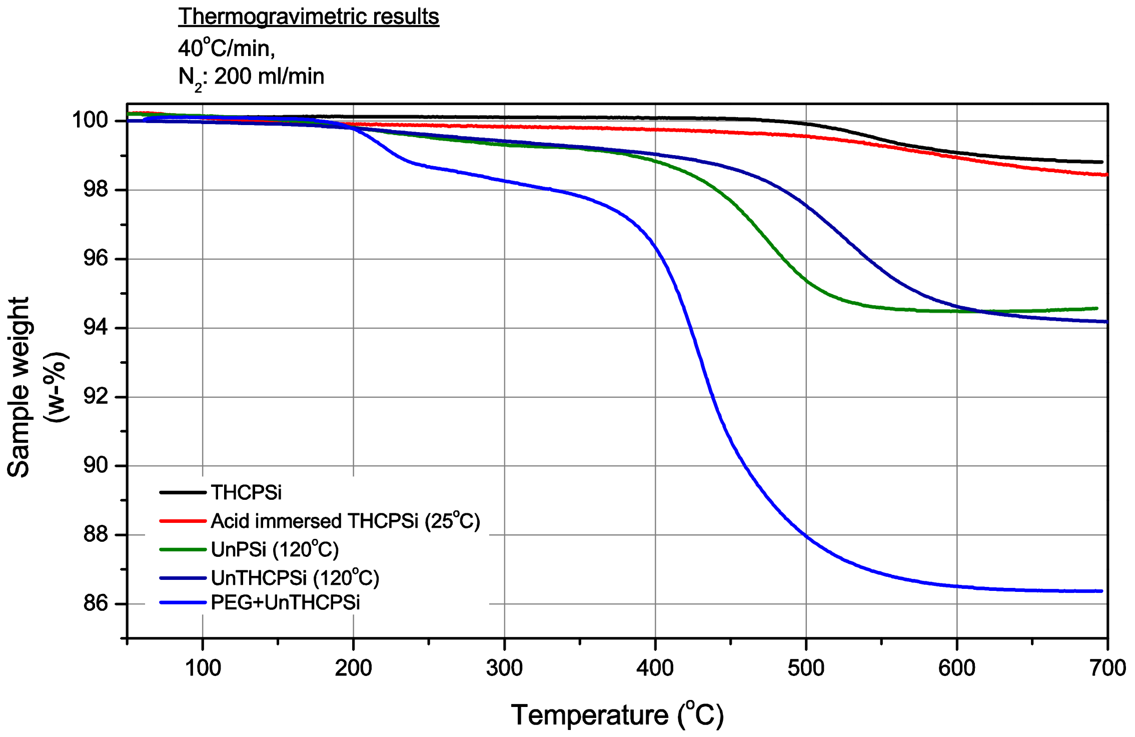

Figure S1. Thermogravimetric (TG) measurements of thermally hydrocarbonized PSi (THCPSi), undecylenic acid treated hydrogen terminated as-anodized PSi (UnPSi) and similarly treated hydrocarbonized PSi (UnTHCPSi). In addition, TG-curves for UnTHCPSi functionalized with amine-terminated PEG 5000, and THCPSi that has been immersed in undecylenic acid at 25°C for 16 hours and washed copiously afterwards. Reduction in weight is related to thermal decomposition of surface moieties desorbing from the surface, and gives qualitative information about the processes involved.

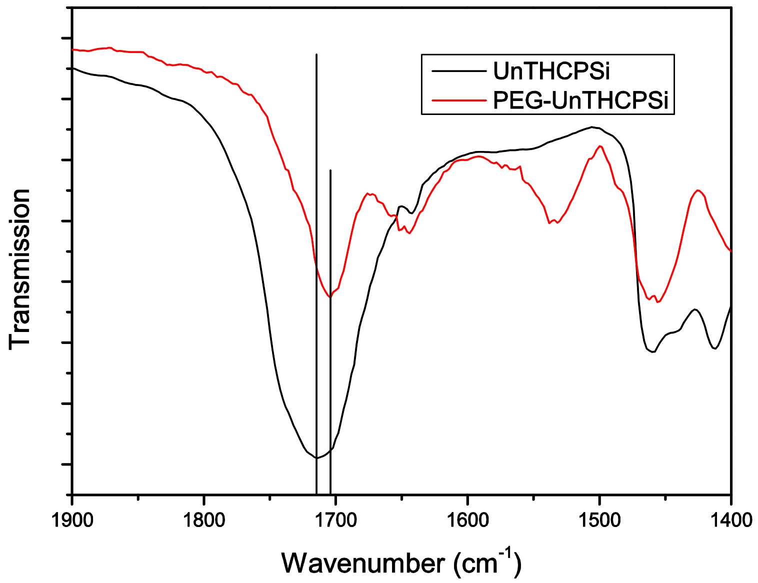

Figure S2. FTIR spectra for UnTHCPSi and UnTHCPSi functionalized with NH_2-PEG 5000. The spectra reveal a shift in the peak related to the carboxylic end of the undecylenic acid molecyle (indicated by the vertical lines). Also the amide bands at 1650 cm^{-1} and 1540 cm^{-1} are clearly visible in the functionalized sample.
